# Vertucci’s root canal configuration of 11,376 mandibular anteriors and its relationship with distolingual roots in mandibular first molars in a Cantonese population: a cone-beam computed tomography study

**DOI:** 10.1186/s12903-022-02078-5

**Published:** 2022-04-16

**Authors:** Yeqing Yang, Chong Jiang, Ming Chen, Junkai Zeng, Buling Wu

**Affiliations:** 1 Stomatological Hospital of Southern Medical University, Guangzhou, People’s Republic of China; 2 School of Stomatology, Southern Medical University, Guangzhou, People’s Republic of China; 3 Guangdong Provincial People’s Hospital, Guangdong Academy of Medical Sciences, Guangzhou, People’s Republic of China; 4 Shenzhen Stomatology Hospital (Pingshan), Southern Medical University, 143 Dongzong Road, Pingshan District, Shenzhen, 518118 People’s Republic of China

**Keywords:** Cone-beam computed tomography, Mandibular anteriors, Distolingual roots, Vertucci’s root canal configuration

## Abstract

**Background:**

Cone-beam computed tomography (CBCT) was used to study the root canal system of mandibular anteriors (MAs) in a Cantonese population and to evaluate the correlation between the complicated root canal configurations of mandibular lateral incisors (MLIs) and the presence of distolingual roots (DLRs) in mandibular first molars (MFMs).

**Methods:**

A total of 11,376 mandibular anterior teeth were scanned by CBCT. Those whose images met the inclusion criteria were first analysed according to Vertucci's root canal configuration and then grouped based on gender, age, and side, and their effects on root canal morphology were analysed. Finally, statistical analysis was used to evaluate the correlation between the complicated root canal configurations of MLIs and the existence of DLRs in MFMs. All statistical analyses were performed by using SPSS 25.0 software. Quantitative data are presented as the mean ± standard deviation. Student’s t tests were used to calculate statistical significance. *P* < 0.05 was considered statistically significant.

**Results:**

In MAs in the Cantonese population, all mandibular central incisors (MCIs) and MLIs had one root, and 0.37% of mandibular canines (MCs) had two roots. The most common Vertucci's root canal configuration was Vertucci I followed by Vertucci III. A total of 30.91% of MLIs and approximately 8% of MCIs and MCs have complicated root canal configurations. There were no significant differences in the prevalence of DLRs in MFMs and the incidence of complicated root canal configurations of MLIs between males and females or between the right and left teeth. However, a significant difference was found in different age groups of root canal configurations in MLIs. Moreover, significant ipsilateral and contralateral correlations between MFMs with DLRs and MLIs with complicated root canal configurations were observed on both sides.

**Conclusion:**

In Cantonese population, the possibility of complicated root canal configuration in MLIs was higher, when DLR appeared in MFMs.

## Background

Successful root canal treatment is very challenging because of increased difficulties in cleaning, shaping, and filling the root canal system in a 3-dimensional manner [1–3]. A comprehensive understanding of the complexity of the root canal system is necessary for successful endodontic treatment. Considering that ethnic factors will affect root canal configuration, it is necessary to study different populations around the world [4].

Mandibular first molars (MFMs) are the first permanent molars to grow into the mouth. Because of pulpitis and periapical periodontitis, root canal treatment is often needed [5]. The root canal system of the MFM is one of the most complicated among human teeth. For example, the system is characterized by a c-shaped root canal [6], distolingual roots (DLRs) [7], middle mesial canals and the occurrence of fusion roots [8]. The DLR, also known as radix entomolaris, is one of the major variants in MFMs [9–12]. The complexity of the root and canal morphology of MFMs can be attributed to ethnicity, age, gender, and study design [13]. Even if the person has partial edentulism, the long-standing teeth in the mouth are still mandibular permanent incisors [14]. Many clinicians mistakenly believe that the mandibular anteriors (MAs) all have single root canals. However, the root canal configurations of MAs may vary greatly with ethnicity and gender. In particular, a MA may have inter-canal communication, additional canals and multiple canal configurations, which may have a key impact on endodontic treatments [15]. Based on the population studied, CBCT studies have demonstrated a high prevalence of second canals in mandibular central incisors (MCIs) and mandibular lateral incisors (MLIs). Second canals were found in approximately 15% of the population in Iran and Turkey [16, 17]. The prevalence of two root canals in MAs in Brazilians was approximately 20% for MCIs and MLIs and 10% for mandibular canines (MCs). Another study in the Chinese population showed that the prevalence for MCIs was similar, but the prevalence for MLIs was higher (27%) [18]. The Malay ethnic group showed more canal variations in MAs than the Chinese and Indian groups [19].

Since 1990, cone-beam computed tomography (CBCT) has been used in the field of endodontics [20]. It provides a noninvasive technique to more accurately assess the root canal system and helps to perform detailed research on internal anatomy. Compared with the periapical, panoramic and micro CT techniques, it has the advantages of high image resolution, 3D reconstruction, and simple operation [21]. CBCT images can be analysed by computer and simultaneously displayed in the sagittal, coronal and axial planes, which is particularly suitable for oral clinical needs.

Although many authors have studied MLIs using CBCT, few studies have reported on the morphological classification in a Cantonese population. In this study, 11,376 MAs were observed by CBCT. To our knowledge, compared with previous studies, our sample size is larger. Additionally, there were few studies on the anatomical symmetry of the left and right mandibular anterior teeth of the same patient, but when treating the contralateral teeth of the same patient, the contralateral teeth have high clinical relevance [22] The presence of DLR is related to many morphologic features, such as crown dimension [23], interorifice distance of canals [10], and distance from buccal cortical bone [24]. However, in previous studies, a possible association between the prevalence of DLR in MFM and the morphological characteristics of other teeth has been investigated. The cooccurrence of DLRs in MFMs and the complicated root canal morphology in MLIs are prominent among Taiwanese patients [25]. However, there is no research report among Cantonese. The main purpose of this study was to provide an in vivo assessment of the prevalence of the different Vertucci's root canal configurations in mandibular anterior teeth in a Cantonese population. CBCT images were used to determine the correlation between the root canal configurations of MLIs and the appearance of DLR in MFMs.

## Methods

### Patients

CBCT images from 2356 Cantonese patients in this study were obtained between January 2018 and January 2019 using the CBCT imaging system and collected from the database of the Department of Oral Radiology, Nanfang Hospital, Southern Medical University, Guangzhou. All patients required radiographic examination of CBCT as part of their dental treatment. The images were taken as part of the routine examination, diagnosis, and treatment planning for patients who included those suffering facial trauma or maxillary sinusitis, who required oral surgery or orthodontic treatment or who needed implant treatment. This project and protocol were approved by the Medical Ethics Committee of Nanfang Hospital (NFEC-2020-106) and conducted in strict accordance with the Declaration of Helsink (2013). CBCT images were obtained using a Planmeca Romexis3D (3D Plus) CBCT scanner (Planmeca, Finland). Board-certified radiologists operated the X-ray tube at an accelerated potential with a field-of-view size (FOV) of 8 × 8 cm, a peak voltage of 84 kV, a beam current of 14 mA and an exposure time of 12 s for a full arch. The voxel size was 200 µm × 200 µm, and the minimum layer thickness was 0.15 mm. A total of 1896 patients' images qualified for further analysis based on the following inclusion criteria:

1. The mandibular central incisors, mandibular lateral incisors, mandibular canines, mandibular first molars were present bilaterally with complete root formation
2. Absence of root canal treatment
3. Complete root formation without evident resorption process
4. Absence of periapical disease
5. Absence of coronal or post and core restorations, which may obscure the imaging study
6. Good quality CBCT images that are clear and free of artefacts.

If the image did not meet the above inclusion criteria, the image needed to be excluded.

### Morphologic analysis and classification

**Fig. 1:**
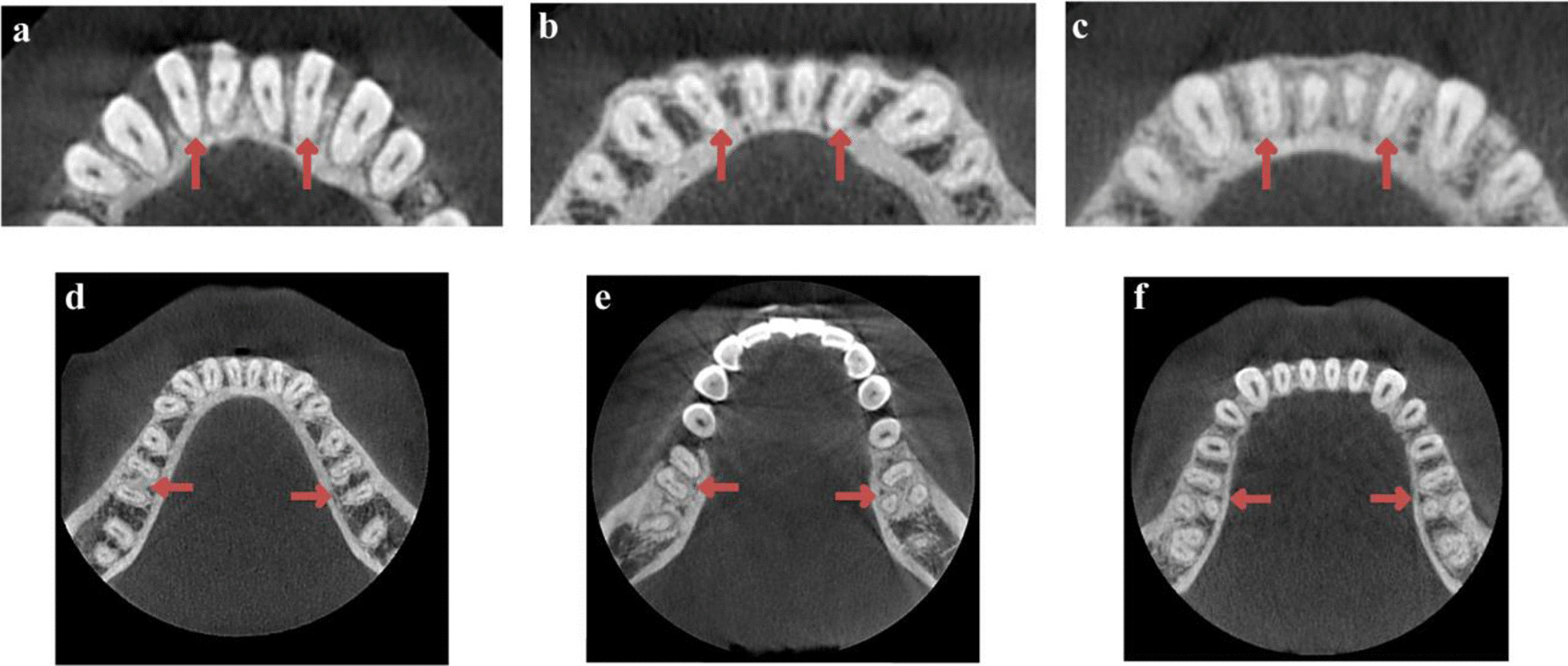
Representative images of root canal configurations of MLIs and the presence or absence of DLRs in MFMs. **a** Both MLIs were single root canal. **b** Right side or left side was single root canal. **c** Both MLIs were complicated root canals. **d** DLRs were found in both MFMs (Bil-DLR). **e** DLR was found in right side or left side MFM (Uni-DLR). **f** No DLR was found in both MFMs (Non-DLR)

The image data were exported in DICOM format. The CBCT images were scanned and analyzed using the Romexis Viewer software from CBCT manufacturer. Calibration for the study was performed between the two endodontists. The observer was trained and calibrated for reading the CBCT images with a sample size of 55. The observer evaluated the CBCT images using axial, sagittal and coronal views to identify root canal morphology. The kappa value is 0.830 (*P* < 0.05). In cases of disagreement, these two endodontists discussed the data until a consensus was reached. An oral radiologist provided guidance when necessary.The presence or absence of DLRs in MFMs was defined according to previous studies [25, 26] (Fig. 1).

1. Non-DLR: the patient has MFMs without DLRs
2. Unilateral DLR (Uni-DLR): the patients has DLR only on the left or right MFM and no DLR on the other side
3. Bilateral DLR (Bil-DLR): the patients has DLRs on both left and right MFMs

Based on previous studies on the MLIs, we classified them into the following categories [25, 26] (Fig. 1):

1. Single: a single canal, with only one canal from the pulp orifice to the apical in MLIs.
2. Complicated: complicated canal, with more than one canal from the pulp orifice to the apical in MLIs.

The single or complicated canal in MLIs was further categorized as follows:

1. Unilateral: the patients had a"single canal"or"complicated canal" in either the left or right MLIs
2. Bilateral: the patients had a"single"or "complicated canal"in MLIs bilaterally

**Fig. 2:**
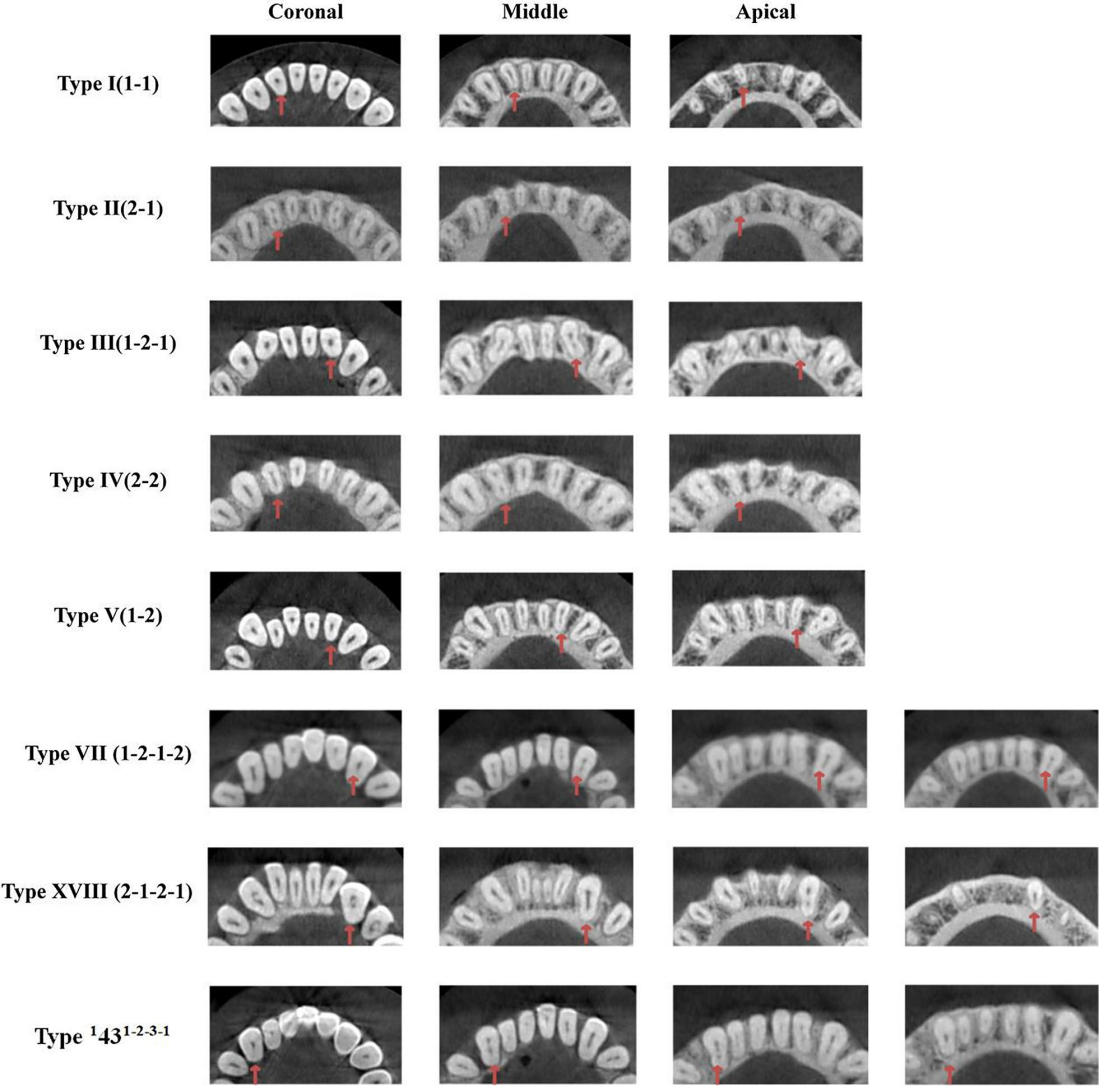
The root canal configuration of MAs were classified according to the Vertucci classification, Vertucci Supplementary classification and a new classification system given by Karobari et al.

In CBCT images, the root canal system of MLIs was classified based on classic Vertucci classification [5], Vertucci Supplementary classification [27] and a new classification system given by Karobari et al. [28] (Fig. 2).

1. Type I (1-1): Pulp chamber bottom have one root canal orifices, and always have one independent root canal
2. Type II (2-1): The pulp chamber bottom has two root canal orifices. Then, these orifices merge into one canal at a certain position of the root canal to yield one apical foramens.
3. Type IlI (1-2-1): The bottom of the pulp chamber has one root canal orifices that branch into two separate root canals. These canals finally merge into one root canal.
4. Type IV (2-2): Two separate canals extend from the pulp chamber to the apical.
5. Type V (1-2): The pulp chamber has one canal and divides short of the apical into two separate canals, with separate apical foramens.
6. Type VI (2-1-2): The pulp chamber has two separate canals, join at the midpoint, and then divide again into two with two separate apical foramens.
7. Type VlI (1-2-1-2):At the bottom of the pulp chamber, there is one root canal orifice that branch into two root canals, fuse into one root canal, and finally emerge through two different apical holes out of the root canal system.
8. Type VIlI (3-3): Pulp chamber bottom have three root canal orifices, and always have three independent root canals, and finally there are three different apical foramens.
9. Type XVIII (2-1-2-1): Two root canals are located at the beginning of the pulp chamber and subsequently merges into one root canal, then branches into two independent root canals. Finally, the same apical foramen is formed [27].
10. Type ^1^43^1-2-3-1^: Only one root canal orifice is located at the bottom of the pulp chamber. The orifice branches into two independent root canals, subsequently divides into three independent root canals, and finally merges into the same root canal at the apex part of the root canal [28].

The contralateral teeth were compared for symmetry of the root canal configuration. The influence of gender on the canal configuration was also analyzed.

### Statistical analysis

Statistical analysis was performed using SPSS (Version25.0; SPSS, Inc, Chicago, IL),a statistical software package for Windows. The measurement data were expressed as the mean standard deviation or percentages as appropriate for each measurement calculated at the individual and tooth levels. The chi-square test was used to analyze differences among categorical variables, such as age(age < 45 vs. age ≥ 45 years), gender(male vs. female), and side(left vs. right) and DLR group(Non-DLR, Uni-DLR, or Bil-DLR). The mean is compared using t tests with significance set at *P* < 0.05.

## Results

Of the 2356 patient CBCT images initially scanned, 1896 patients (3792 MLIs and 3792 MFMs) were eligible for further analysis. Of these subjects, 1016 (53.59%) were females and 880 (46.41%) were males, with an average age of 41.22 ± 12.23 years and 40.56 ± 14.56 years, respectively (Table 1).

**Table 1:** Characteristics of the study Cantonese population of the teeth

	Number of patients	Age(years)			Number of teeth (n)	
	n(%)	Mean ± SD	Max	Min	MLIs	MFMs
Female	1016(53.59%)	41.22 ± 12.23	72	18	2032	2032
Male	880(46.41%)	40.56 ± 14.56	70	18	1760	1760
Total	1896(100.00%)	40.86 ± 13.77	72	18	3792	3792

A total of 11,376 MCIs, MLIs and MCs were assessed. Based on Vertucci's root canal configuration (1984), Vertucci Supplementary classification and a new classification system given by Karobari et al, the distribution of teeth is shown in Table 2. Among them, the most common root canal type for MAs was Vertucci type I at 84.65% (9630/11376), followed by Vertucci type III at 14.39% (1637/11376): 7.60% (288/3792) of MCIs, 7.54% (286/3792) of MCs, and 30.91% (1172/3792) of MLIs were complicated root canal configurations. In addition, we also discovered a new root canal type of the MC, which was type Type ^1^43^1-2-3-1^ (Table 2). All MCIs and MLIs had only one root, and 0.37% (14/3792) of MCs had two roots (Table 3).

**Table 2:** Incidence of different root canal morphologies of mandibular anterior

Vertucci’s classification	Number of specimens n(%)					
	33	32	31	41	42	43
Type I(1-1)	1750 (92.30%)	1312 (69.20%)	1754 (92.51%)	1750 (92.30%)	1308 (68.99%)	1756 (92.62%)
Type II(2-1)	2 (0.11%)	6 (0.32%)	0 (0.00%)	0 (0.00%)	6 (0.32%)	0 (0.00%)
Type III(1-2-1)	124 (6.54%)	554 (29.22%)	136 (7.17%)	140 (7.38%)	554 (29.22%)	129 (6.80%)
Type IV(2-2)	0 (0.00%)	0 (0.00%)	0 (0.00%)	0 (0.00%)	2 (0.11%)	0 (0.00%)
Type V(1-2)	16 (0.84%)	24 (1.27%)	6 (0.32%)	6 (0.32%)	26 (1.37%)	8 (0.42%)
Type VI(2-1-2)	0 (0.00%)	0 (0.00%)	0 (0.00%)	0 (0.00%)	0 (0.00%)	0 (0.00%)
Type VII(1-2-1-2)	2 (0.11%)	0 (0.00%)	0 (0.00%)	0 (0.00%)	0 (0.00%)	0 (0.00%)
Type VIII (3-3)	0 (0.00%)	0 (0.00%)	0 (0.00%)	0 (0.00%)	0 (0.00%)	0 (0.00%)
Type XVIII (2-1-2-1)	2 (0.11%)	0 (0.00%)	0 (0.00%)	0 (0.00%)	0 (0.00%)	2 (0.11%)
Type ^1^43^1-2-3-1^	0 (0.00%)	0 (0.00%)	0 (0.00%)	0 (0.00%)	0 (0.00%)	1 (0.05%)
Total	1896	1896	1896	1896	1896	1896

Type ^1^43^1-2-3-1^ was an additional type; 31, left mandibular central incisor; 41, right mandibular central incisor; 32, left mandibular lateral incisor; 42, right mandibular lateral incisor; 33, left mandibular canine; 43, right mandibular canine

**Table 3:** Incidence of the number of roots of mandibular anterior

Number of roots	Number of specimens n(%)					
	33	32	31	41	42	43
1	1886 (99.47%)	1896 (100.00%)	1896 (100.00%)	1896 (100.00%)	1896 (100.00%)	1892 (99.78%)
2	10(0.52%)	0(0.00%)	0(0.00%)	0(0.00%)	0(0.00%)	4(0.21%)

**Fig. 3:**
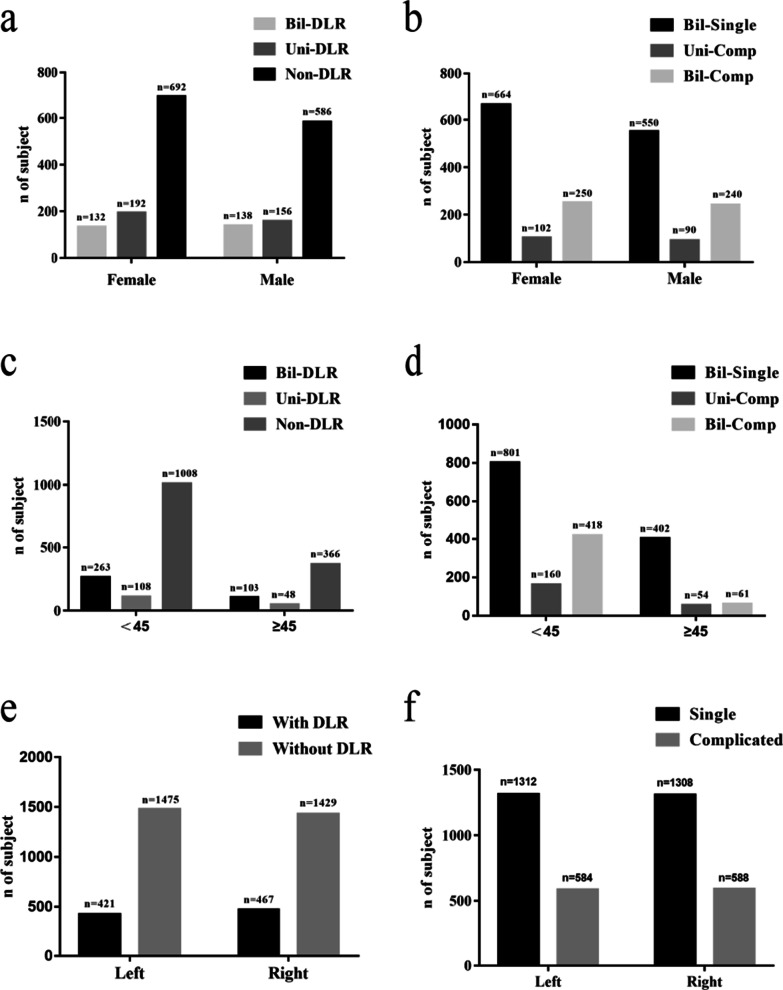
Characteristics of the study population examined teeth by genders, age groups, and sides. **a** The distribution of MFMs with DLRs between males and females. **b** The distribution complicated root canal configurations of MLIs between males and females. **c** The distribution of MFMs with DLRs between different age groups. **d** The distribution complicated root canal configurations of MLIs between different age groups. **e** The distribution of MFMs with DLRs between sides. **f** The distribution complicated root canal configurations of MLIs between sides

At the patient-level analysis, among 1016 females, bilateral complicated (Bil-Comp) root canals and bilateral single (Bil-Single) root canals were found in 65.35% (664/1016) and 24.61% (250/1016) of teeth, and 10.04% (102/1016) of the patients had inconsistent root canal configurations. Among 880 males, Bil-Comp root canals and Bil-Single root canals were found in 62.50% (550/880) and 27.27% (240/880) of teeth, and 10.23% (90/880) of patients had inconsistent root canal configurations. The prevalence of Bil-DLR and Uni-DLR in MFMs was 13.00% (132/1016) and 18.90% (192/1016), respectively, in the female group. In the male group, the prevalence of Bil-DLR and Uni-DLR in MFMs was 15.68% (138/880) and 17.73% (156/880), respectively (Fig. 3). There were no significant differences in the prevalence of DLRs in MFMs (*P* = 0.13) or the incidence of complicated root canal configurations of MLIs (*P* = 0.06) between males and females (Table 4).

**Table 4:** Characteristics of the study population examined teeth by genders, age groups, and sides

	Number of specimens (n)			Number of specimens (n)		
	MLI			MFM		
	Single	Complicated	*P*-value	Without DLR	With DLR	*P*-value
Total	2620	1172		2904	888	
Sex						
Female	1430	602	*P* = 0.06	1576	456	*P* = 0.13
Male	1190	570		1328	432	
Side						
Left	1312	584	*P* = 0.89	1475	421	*P* = 0.07
Right	1308	588		1429	467	
Age						
< 45	1762	996	****	2124	634	*P* = 0.31
≥ 45	858	176		780	254	

The level of statistical significance was set at *P* < 0.05. ****P < 0.0001

In addition, there were no significant differences in the prevalence of DLRs in MFMs (*P* = 0.07) or the incidence of complicated root canal configurations in MLIs (*P* = 0.89) between the left and right sides. We came to this result by using statistical methods (Table 4). In subjects with complicated MLIs, 30.80% (584/1896) were found in the left group and 31.01% (588/1896) in the left group; moreover, of the DLRs in MFMs, 22.20% (421/1896) were found in the left group and 24.63% (467/1896) were found in the right group (Fig. 3).

There were significant differences in the prevalence of complicated root canal configurations in MLIs between age groups (age < 45 years vs. age ≥ 45 years, *P* < 0.0001) (Table 4). The prevalence of Bil-DLR and Uni-DLR in MFMs was 19.07% (263/1379) and 7.83% (108/1379), respectively, in the age < 45 years group. In the age ≥ 45 years group, the prevalence of Bil-DLR and Uni-DLR in MFMs was 19.92% (103/517) and 9.28% (156/517), respectively (Fig. 3). There were no significant differences in the prevalence of DLRs in MFMs (*P* = 0.13) or the incidence of complicated root canal configurations of MLIs (*P* = 0.06) between males and females (Table 4).

Among the bilateral MFMs, 83.97% (1592/1896) either both had or both did not have DLRs. Among the bilateral MLIs, 85.97% (1630/1896) had complicated root canal configurations on either both sides or neither side. These results reflect the high symmetry of the root canal morphology across bilateral teeth in the Cantonese population. In addition, regardless of whether the ipsilateral side or the contralateral side was examined, there was a correlation between the existence of DLR of the MFM and the complicated root canal configurations of the MLI (Table 5).

**Table 5:** Analysis of the correlation of MFMs with DLRs and root canal configurations of MLIs ipsilaterally and contralaterally

	Number ofspecimens (n)							
	MFM, Right							
	Without DLR	With DLR	Total	*P*-value				
MFM, Left				****				
Without DLR	1300	175	1475					
With DLR	129	292	421					
Total	1429	467	1896					

	Number of specimens (n)							
	MLI, Right							
	Single	Complicated	Total	*P*-value				
MLI, Left				****				
Single	1177	135	1312					
Complicated	131	453	584					
Total	1308	588	1896					

	Number of specimens (n)				Number of specimens (n)			
	MLI, Left				MLI, Right			
	Single	Complicated	Total	*P*-value	Single	Complicated	Total	*P*-value
MFM, Left								
Without DLR	1050	425	1475	***	1055	420	1475	****
With DLR	262	159	421		253	168	421	
Total	1312	584	1896		1308	588	1896	
MFM, Right								
Without DLR	1017	412	1429	**	1011	418	1429	**
With DLR	295	172	467		297	170	467	
Total	1312	584	1896		1308	588	1896	

The level of statistical significance was set at *P* < 0.05. **P < 0.01, ***P < 0.001, ****P < 0.0001

## Discussion

Knowledge of root canal anatomy is essential for successful root canal treatment. Endodontic treatment of mandibular anterior teeth and mandibular first molar treatments can be challenging in the presence of additional root canals, which may not be recognized by periapical radiographs. Previous literature has reported a positive correlation between missing root canals and persistent apical periodontitis [2, 3, 29]. CBCT may be considered if the clinician suspects the presence of multiple root canals. The accuracy of CBCT is much higher than that of periapical radiographs [30, 31]. 

Carabelli first referred to DLR in 1884, often describing it as the presence of an additional lingual root and the major anatomical variation in MFM [9–12, 23, 32, 33]. Previous studies have reported that the presence of this dental trait has been thought to be a genetically determined racial trait. In whites and blacks, the frequency of DLR is less than 5%, while in populations with ethnic Mongolian characteristics (for example, Chinese, Japanese, Korean, and Taiwanese), the frequency of DLR ranges from 5 to 30% [34–36].

The complex anatomical variation in MLIs makes it one of the most difficult teeth for root canal therapy [37, 38]. In this study, we evaluated the frequency of the Vertucci root canal configuration and the gender influence on its distribution in 11,376 mandibular anterior teeth in a Cantonese subpopulation. This is a large sample size study. More than 3792 teeth of each type (MCI, MLI or MC) were evaluated. Finally, we evaluated the correlation between the root canal configuration of MLI and the appearance of DLR in MFM.

This study found that all MCIs and MLIs had single roots, which was consistent with previous studies conducted in Brazil [39], China [18] and Iran [40]. However, some studies have found that MLIs with two roots has been found in Turkish [17] and Chinese [41] populations, but the prevalence is low (0.1% and 0.3%, respectively). In addition, the prevalence of two root MCs has been reported in multiple studies and ranges from 0.8 to 4.7% depending on the ethnic groups and study model. The highest prevalence was reported in the Iranian population (4.7%) [41], followed by Turkish population (3.1%) [17]. East Asian regions have been associated with the lowest prevalence of two rooted MCs, in a Shandong population (1.32%) [18] and a Chongqing population (0.8%) [40]. And two rooted MCs were found in only around 0.37% of cases in our study. The results of this study are lower than those reported in the literature, which may be due to ethnic differences. In our study, the presence of MFMs with DLR was significantly correlated with the complicated root canal configuration in MLIs. In other words, a significant ipsalateral correlation between MFMs with DLRs and MLIs with complicated root canal configuration was observed on both sides (left (*P* < 0.0005) and right (*P* < 0.0001)). At the same time, correlations between MFM with DLR and MLIs with the complicated root canal configuration were also observed on both sides (left (*P* < 0.0015) and right (*P* < 0.004)). These results were different from those reported by Wu et al. in Taiwanese individuals [25], which may be caused by different regions and sample sizes.

Moreover, in this study, no significant differences in the prevalence of complicated root canal configurations in MLIs and the frequency of DLR in MFMs were noted between men and women. However, a higher incidence of complicated root canal configurations was observed in the younger group (age < 45 years, 36.11%) than the older group (age > 45 years, 17.02%) (*P* < 0.0001, Table 4), which was consistent with previous studies [25]. The detection rate of complicated root canal configurations decreased significantly in older group (age > 45 years). This decreased detection rate may not be attributed to a decrease in the actual existence rate of complicated root canal configurations but may be attributed to calcification of the root canal and secondary dentin hyperplasia, which reduced the diameter of the root canal. In view of the resolution of CBCT, it may not be detected [42]. 

Our research results on the morphology of mandibular incisors and the correlation between complicated root canal configurations of different teeth provide necessary knowledge to clinicians: in particular, the bilateral symmetry rate of DLR in MFMs, high levels of complicated canal configurations in MLIs, and a correlation between the two. Therefore, clinicians should be aware that if a patient has complicated canal configurations in MLIs, there may be DLR in the MFMs and complicated anatomical structures in other teeth. However, due to the lack of research data, further studies are needed to investigate the relationships with the other teeth. CBCT has many advantages over other experimental methods to evaluate root canal morphology, such as clearing and staining, sectioning, conventional radiographs, etc., but there were still some limitations to be emphasized when using CBCT to evaluate root canal morphology. For example, it was difficult for CBCT to detect micro structures, such as branches of root canals. In addition, calcified root canals or root canals with extremely narrow diameter may not be detected. Therefore, it would be better to evaluate the relationship between the correlation of MFMs with DLRs and complicated root canal configurations in MLIs by using a more accurate method, like micro computed tomography.

## Conclusion

Among the MAs in the Cantonese population, the presence of complicated root canal configurations in MCIs and MCs were 8% and MLIs were 30%. More than 80% of the MAs and the contralateral teeth have symmetrical root canals. The canal systems of MLIs were varied and complex, the incidence of complicated root canal configurations in MLIs declined as age increased. Moreover, there was no significant difference in the incidence of complicated root canal configurations between the right and left MLIs in both males and females. At the same time, this study showed that when there were DLRs in MFMs in the Cantonese population, the possibility of complicated root canal configuration in MLIs was higher. However, as far as we know, there are few research articles on the correlation between MFMs with DLRs and complicated root canal configurations in MLIs, so we need to study the differences between different ethnicity of the patients and the correlation between different teeth in the future. But clinicians must not only be aware of the changes in the number of roots and root canal configurations but also be aware that the anatomical differences between teeth may vary and be correlated.

## Data Availability

The datasets used and/or analysed during the current study are available from the corresponding author on reasonable request.

## Abbreviations

CBCT: Cone-beam computed tomography
MA: Mandibular anterior
MLI: Mandibular lateral incisor
DLR: Distolingual root
MFM: Mandibular first molar
MCI: Mandibular central incisors
MC: Mandibular canine
Bil-Comp: Bilateral complicated
Bil-Single: Bilateral single
Bil-DLR: Bilateral DLR
Uni-DLR: Unilateral DLR
